# A Field-Deployable Insulated Isothermal PCR (iiPCR) for the Global Surveillance of *Toxoplasma gondii* Infection in Cetaceans

**DOI:** 10.3390/ani12040506

**Published:** 2022-02-17

**Authors:** Meng-Jung Hsieh, Wei-Cheng Yang

**Affiliations:** School of Veterinary Medicine, National Taiwan University, Taipei 10617, Taiwan; nono8139@gmail.com

**Keywords:** insulated isothermal PCR, cetaceans, *Toxoplasma*, pollution, field-deployed

## Abstract

**Simple Summary:**

Since high trophic levels marine mammal species share the coastal environments and diets with humans, cetaceans provide an indication of contaminant bioaccumulation in humans and may serve as sentinels for public health problems. Parasite monitoring in marine sentinels can assist in evaluating the quality of the aquatic ecosystem’s health. *T. gondii* infection in cetaceans is an indicator of land-to-sea coastal pollution. Although *T. gondii* infection cases in cetaceans have been reported in several countries, an information gap still exists in some areas. The present study employs a portable insulated isothermal PCR (iiPCR) with an automatic extraction device as a rapid, affordable, user-friendly, and field-deployable platform to rapidly detect nucleic acid of *T. gondii* in stranded cetaceans. The platform utilizes duplex iiPCR designed to simultaneously detect *T. gondii* and a housekeeping gene of cetacean on the samples, which can prevent the false-negative results of pathogen detection and improve the accuracy of surveillance. This study would contribute to improving the environment through the warning of the sentinel animals and building new strategies by detecting the occurrence of land-based biological pollution.

**Abstract:**

Toxoplasmosis is a zoonotic disease with veterinary and public health importance worldwide. *Toxoplasma gondii* infection in cetaceans is an indicator of land-to-sea oocyst pollution. However, there is a critical knowledge gap within the distribution of the *T. gondii* infection in cetaceans. To facilitate the global surveillance of this important zoonotic pathogen, we developed a field-deployable duplex insulated isothermal PCR (iiPCR) with automated magnetic bead-based DNA extraction for the on-site detection of *T. gondii* in stranded cetaceans. It targets the B1 gene of *T. gondii* combined with β2-microglobulin (B2M) gene of cetaceans as an internal control. Compared with the conventional qPCR assay, B1/B2M duplex iiPCR assay showed comparable sensitivity (21~86 bradyzoites in 25 mg of tissue) to detect spike-in standard of *T. gondii* DNA in cerebrum, cerebellum, skeletal muscle and myocardium tissues. Moreover, the overall agreement between the duplex iiPCR and qPCR was in almost perfect agreement (92%; 95% CI: 0.78–0.90; κ = 0.84) in detecting a synthetic spike-in standards. The B1/B2M iiPCR assay coupled with a field-deployable system provides a prompt (~1.5 h), feasible, highly sensitive and specific on-site diagnostic tool for *T. gondii* in stranded cetaceans. This platform provides one approach to evaluating aquatic ecosystem health and developing early warnings about negative impacts on humans and marine animals.

## 1. Introduction

**Figure 1:**
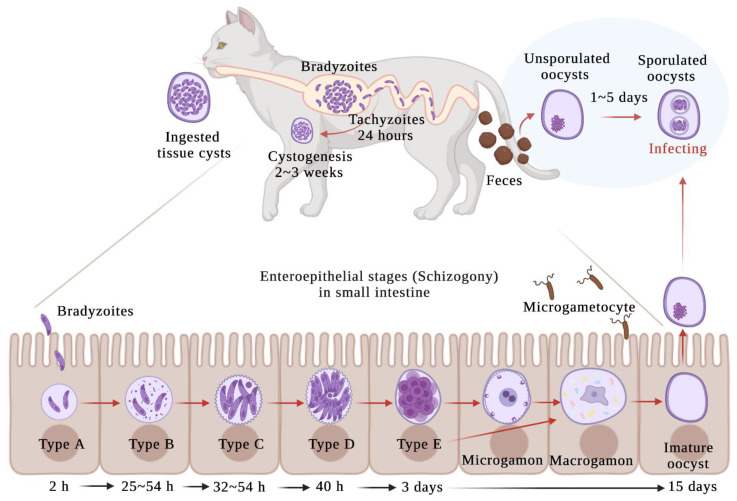
Enteroepithelial stages of *T. gondii* in epithelial cells of the small intestine from a domestic cat. Schematic based mainly on Moura et al., 2009.

*Toxoplasma gondii* is well-studied because of its medical and veterinary importance. *T. gondii* is an intracellular parasite, a widespread zoonotic protozoan, which infects all warm-blooded animals [1]. *T. gondii* can be transmitted by a fecal-oral cycle, transplacentally, and by carnivorism. Members of the Felidae are the only known definitive host that supports sexual multiplication and sheds infective oocysts into the environment [2,3]. There are three stages of *T. gondii* that are infectious for all hosts: tachyzoites, bradyzoites, and oocysts [4,5]. When an intermediate host consumes the oocysts, the tachyzoites spread from the oocysts and become a rapidly replicating stage in the acute infection. Toxoplasmosis may cause by the tachyzoites in the intermediate host. The immune system does not eliminate tachyzoites, and some of them convert to the slowly growing bradyzoite stage stored in the brain, muscle, heart, and other tissues of the intermediate host [6]. Bradyzoites, which are in a slow replication stage, are contained in tissue cysts and establish a chronic infection, demonstrating a balance between host and parasite. After being consumed by felids, bradyzoites enter the enteroepithelial cells of felids and enter the sexual stage. Enteroepithelial stages (Figure 1) of *T. gondii* (types A, B, C, D and E) occurs only in felids, resulting in the formation of immature oocysts [7]. Unsporulated oocysts are shed in the felids’ feces for approximately 2 weeks. After shedding, parasite oocysts take 1–5 days to sporulate in the environment and become infective [8]. 

**Figure 2:**
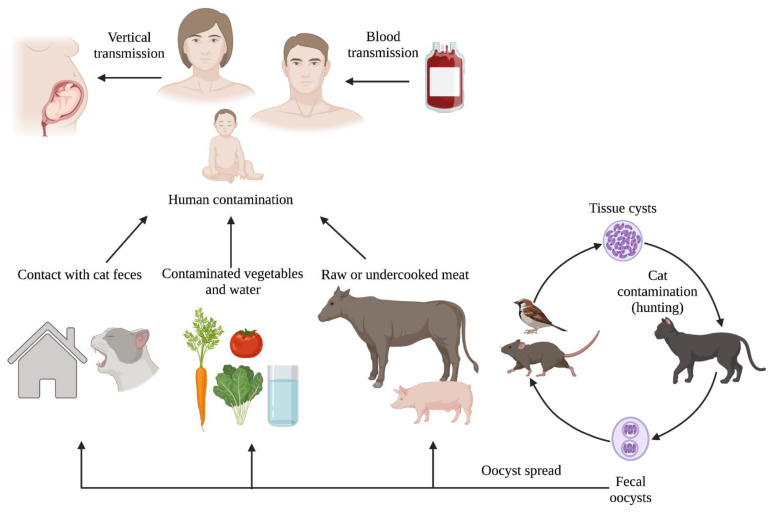
Major routes of transmission of *T. gondii.* Schematic based mainly on Esch and Petersen, 2013.

Recently, *T. gondii* was found to cause neurological symptoms in many species. Clinical signs are rare in infected cats, but clinical toxoplasmosis has been documented in some immunosuppressed cats [9]. *T. gondii* would abolish the innate fear response of rats for the odor of cat urine, converting this aversion to an attraction [10,11]. This may increase the likelihood of a cat predating a rat and increase parasite transmission through the trophic route. In wild animals, cubs of hyena (*Crocuta crocuta*) infected with *T. gondii* were closer to lions and had a higher mortality rate than uninfected hyenas [12]. California sea otters (*Enhydra lutris nereis*) with toxoplasmosis may be prone to shark predation [13]. In meat-producing animals, tissue cysts of *T. gondii* were observed in infected pigs, sheep, goats, poultry, pigeons, farm deer, game animals (including hares and birds), domestic rabbits, dogs, horses, buffaloes, cattle, chickens, ducks, camels and equines (reviewed in [14,15]). Tachyzoites of *T. gondii* were detected in raw chicken eggs and the milk of sheep, goats, cows, and donkeys (reviewed in [15,16]). Currently, an estimated 2 million people globally are infected with *T. gondii* [17]. The parasite transmission can occur by consuming raw or undercooked meat containing tissue cysts, ingesting food or water contaminated with oocysts, or direct contact with oocysts shed in cat feces (Figure 2). Due to the increasing population of cats, this might present a significant exposure risk of humans and other animals. Cases of clinical toxoplasmosis in wildlife have been discovered in wild rabbits, squirrels, canaries, finches, deer, bears, raccoons, lemurs, squirrels, monkeys, and marmosets (reviewed in [16]). Toxoplasmosis is considered to be an emerging disease that can cause mortality, abortion, and decline in wildlife populations.

**Figure 3:**
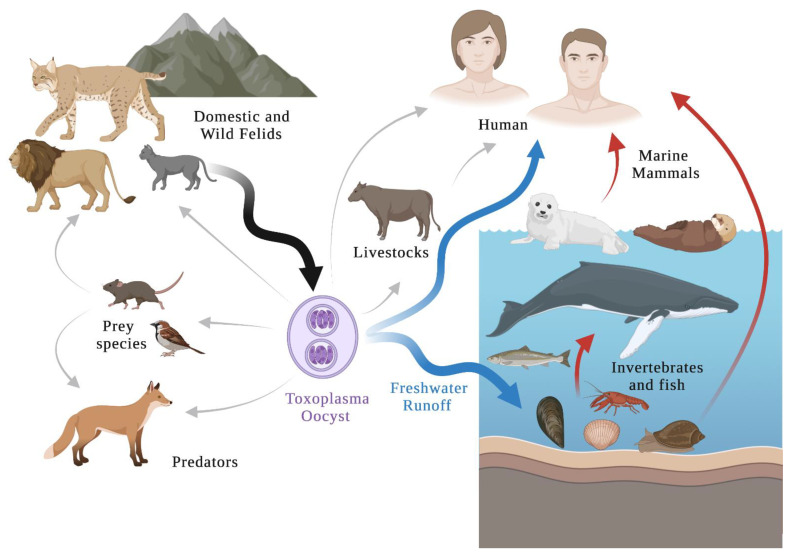
A route of *T. gondii* oocyst-induced transmission. Domestic and wild felids are the only known source (black arrow) of *T. gondii* oocysts. The environmental resistant oocysts can be transported in freshwater runoff (blue arrow). The possible infection routes of terrestrial (gray arrows) and marine species (gray arrows) may be exposed directly to oocysts or indirectly through food sources. Schematic based mainly on https://counterbite.wordpress.com/2019/09/28/cats-and-the-ecosystem/, accessed on December 2021.

Human activities and fecal pollution of captive animals in coastal areas contribute to environmental degradation and lower water quality [18]. The investigation demonstrated that anthropogenically driven habitat degradation has favored the transport of *T. gondii* oocysts to coastal waters, and oocyst-induced infections are recognized as a significant transmission route [19]. Felids can shed millions of *T. gondii* oocysts that spread the infection to many other susceptible hosts. Hardy oocysts can be transported in the freshwater runoff, spreading into the environment [20]. The environmentally resistant oocysts could survive in water for six months [21]. The chemical exposure to neither sodium hypochlorite nor ozone can inactivate *T. gondii* oocysts [22]. Some *T. gondii* oocysts retained their viability even after their exposure to pulsed and continuous UV radiation as evidenced by mouse bioassay [23]. The possible infection routes of terrestrial and marine species may be exposed directly to oocysts or indirectly through food sources. Snails, oysters, mussels, crabs, or other invertebrates can serve as the bioaccumulation of parasitic oocysts, while filter-feeding fishes as biotic vectors for *T. gondii* in the marine environment, which transport oocysts from the nearshore to the pelagic zone [24,25]. These marine animals could be consumed by humans, and might be the source of human toxoplasmosis (Figure 3). These issues have increased the potential exposure for *T. gondii* among ecosystems and wildlife. Detecting the terrestrial parasite in marine animals can be considered an indicator of land-to-sea pollution. As apex predators, marine mammals can also be infected by *T. gondii* by consuming contaminated filter feeders [20].

The first toxoplasmosis case in marine mammals was an infant sea lion reported in 1951 in the United States [26]. After that, *T. gondii* infections were further reported in cetaceans, sirenians, pinnipeds, and sea otters [27]. These marine creatures serve as migratory marine intermediate hosts carrying *T. gondii* tissue cysts and helping to transfer the infection to humans (Figure 3). A serosurvey of Inuit in Nunavik, Quebec, Canada, revealed a *T. gondii* 60% seroprevalence among adults [28]. The most likely source of *T. gondii* in the Inuit people is from the consumption of traditionally prepared foods, including raw meats and organs from intermediate hosts, such as beluga, seal, and walrus [29,30]. Severe toxoplasmosis cases in humans leads to retinochoroiditis and central nervous system disturbances [16]. Central nervous system disturbances might occur in humans, leading to an increased risk of car accidents, mental illness, neuroticism drug abuse and suicides (reviewed in [17]). In immunocompromised individuals, this infection phase can be fatal [14]. Moreover, infections in the first stages of pregnancy can cause abortion, death, or severe fetal damage. It is an important zoonotic disease and public health issue.

**Figure 4:**
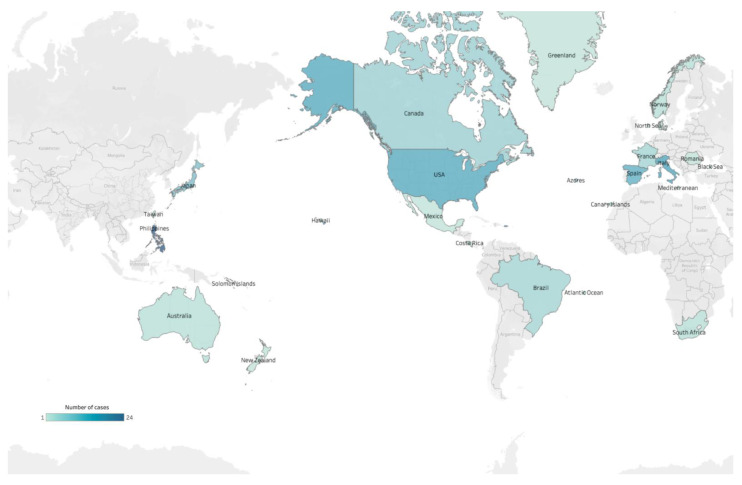
The world map of *T. gondii* or *T. gondii*-like protozoan parasite exposure in cetaceans.

**Figure 5:**
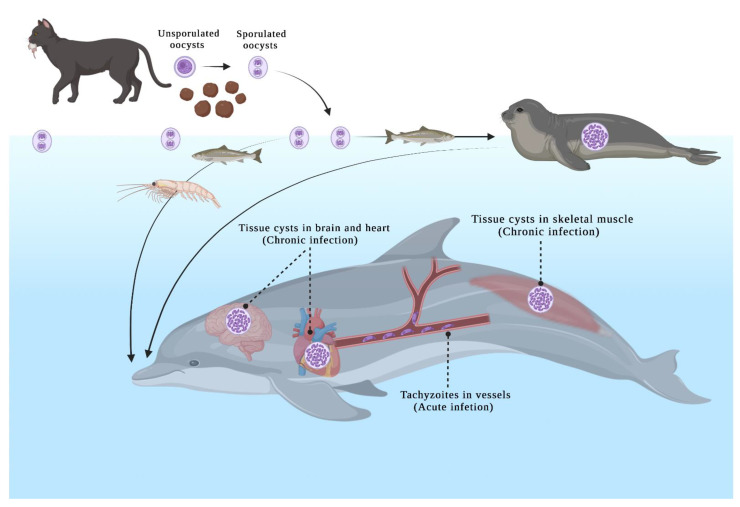
The asexual stage of *T. gondii* in cetaceans after ingesting oocysts or tissue cysts. Unsporulated oocysts are shed in the cat’s feces, and take 1–5 days to sporulate in the environment and become infective. The invertebrates or filter-feeding fish can harbor the oocysts in the pelagic environment.

*T. gondii* or *T. gondii*-like protozoan parasite exposure have been reported in numerous cetacean species (Table 1) (reviewed in [27,31,32,33,34]). These cases were recorded in certain regions, including North America, Western Europe, and Oceania (Figure 4). The asexual stage of *T. gondii* begins after the ingestion of oocysts or the tissue cysts (Figure 5). Studies demonstrated that the toxoplasmosis lesions in cetaceans were non-suppurative meningoencephalitis and choroiditis, lymphoplasmacytic perivascular cuffs, gliosis, neuronal degeneration, necrosis, abortion, and may lead to a decline in health, contributing to stranding and mortality events [25,35,36,37]. Reproduction and population health effects can be huge for threatened populations. Besides, *Toxoplasma gondii*-associated encephalitis was exacerbated by coinfection with *Brucella* spp. and *Listeria monocytogenes* in a wild striped dolphin (*Stenella coeruleoalba*) from Italy [38].

Cetaceans have numerous advantages as marine sentinels, such as long-life spans, and unique fat stores that can serve as depots for anthropogenic toxins [39,40]. These toxins characterize the impacts that ultimately affect animal and human health associated with the oceans. Additionally, parasite monitoring in marine sentinels can assist in evaluating the quality of the aquatic ecosystem’s health. Given the high trophic levels in cetacean species, which share the coastal environments and diets with humans, cetaceans provide an indication of contaminant bioaccumulation in humans and may serve as sentinels for public health problems [41]. *T. gondii* infection in cetaceans could serve as an indicator of land-to-sea coastal oocyst pollution. 

The detection of *T. gondii* infection can be categorized into non-DNA-based and DNA-based diagnostic procedures (reviewed in [42]). Non-DNA-based procedures are conducted through serological tests, which include dye test (DT), indirect fluorescent antibody test (IFAT), modified agglutination test (MAT), latex agglutination test (LAT), indirect hemagglutination (IHA), and enzyme-linked immunosorbent assays (ELISA). The DNA-based procedures include conventional PCR, nested PCR, real-time quantitative PCR (qPCR), loop-mediated isothermal amplification (LAMP), and recombinase polymerase amplification (RPA) [43]. These traditional diagnoses of *T. gondii* were conducted in a well-established laboratory. Although *T. gondii* infection cases in cetaceans have been reported in several countries (reviewed in [27]), an informational gap still exists in some areas, such as Southeastern Asia, South Asia, South America and Africa (Figure 4). This is likely due to less effort in *T. gondii* detection in stranded cetaceans in these areas because of weather conditions, long-distance transportation leading to DNA degradation, or other logistic difficulties in finding an appropriate agency. This should be regarded as a critical knowledge gap in *T. gondii* epidemiology in cetaceans. This study employs a portable insulated isothermal PCR (iiPCR), using an automatic extraction device as a rapid, affordable, user-friendly, and field-deployable platform to rapidly detect nucleic acid of *T. gondii* in stranded cetaceans. This study would contribute to an improvement in the environment by warning of the sentinel animals and building new strategies by detecting the occurrence of land-based biological pollution.

## 2. Materials and Methods

### 2.1. Ethic Statement

All animal procedures were conducted with the approval of the Ocean Conservation Administration (OAC), Taiwan (Permit #090002352).

### 2.2. Synthetic Spike-in Standard

**Figure 6:**
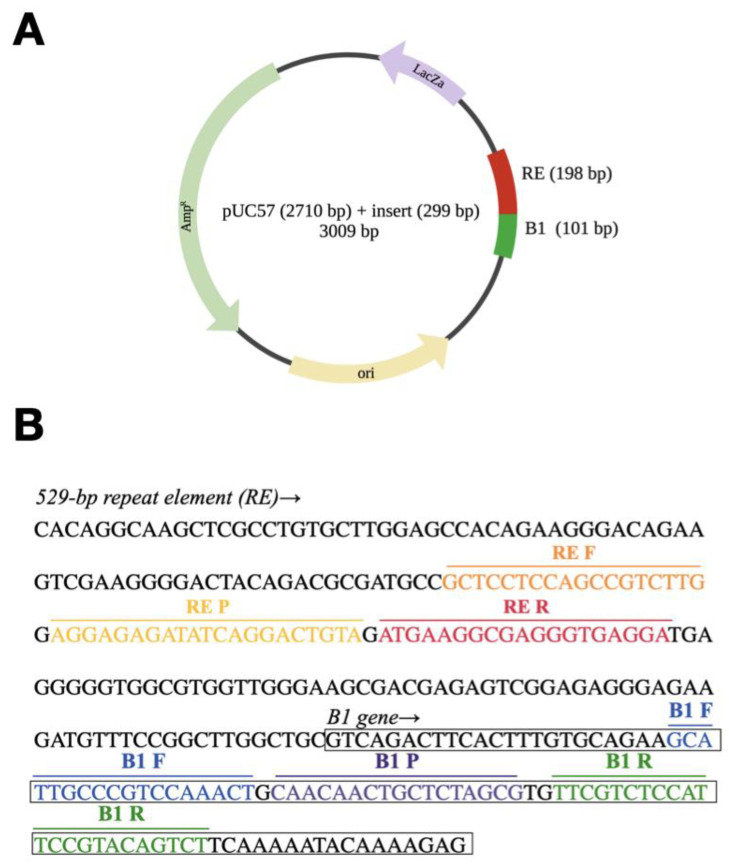
(**A**): The map of synthetic spike-in standard; (**B**): The sequence of synthetic spike-in standard. The sequences were based on the partial sequence of RE and B1 genes. DNA sequences used for primer and probe design are shown by heavy lines.

The sequence of synthetic spike-in standard as shown in figure (Figure 6A,B). The synthetic spike-in standard contained a fragment of the B1 gene (GenBank accession no. AF179871) [44] and a fragment of the 529-bp repeat element (RE) (GenBank accession no. AF146527) [45] of *T. gondii*. They were synthesized on the basis of the pUC57 plasmid (Biotools, New Taipei, Taiwan).

### 2.3. DNA Extraction

**Figure 7:**
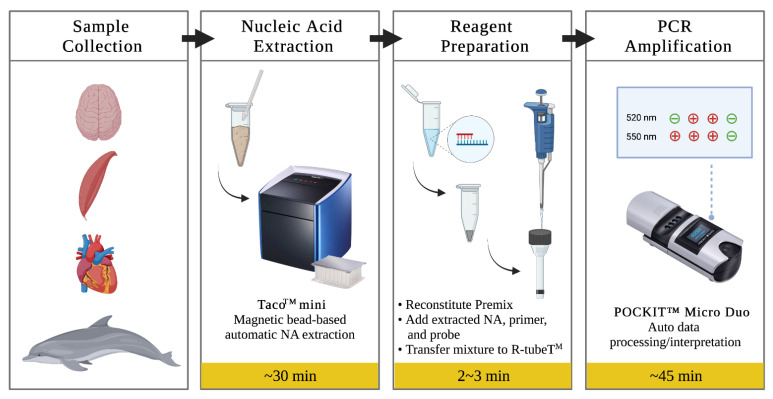
POCKIT™ GeneReach, Lexington, MA, USA) (system workflow for point-of-need detection of *T. gondii* synthetic spike-in standard. This system includes a compact automatic nucleic acid extraction device (taco™ mini) and a portable PCR device (POCKIT™). After sample collection, nucleic acids are extracted using a preloaded extraction plate in approximately 30 min. Subsequently, the lyophilized iiPCR reaction is reconstituted, and nucleic acids are added and tested. The mixture was transferred to an R-tube and tested in a POCKIT device. TaqMan^®^ probe hydrolysis-based amplification signals are detected and automatically processed, providing qualitative results on the display screen after 45 min. One to four reactions could be performed simultaneously in one run. Schematic based mainly on Carossino et al., 2017.

The cerebrum, cerebellum, skeleton muscle, myocardium tissues, which were confirmed to be *T. gondii*-negative by qPCR, were obtained from fresh stranded cetacean carcasses in Taiwan from 2019 to 2021, including *Delphinus delphis*, *Globicephala macrorhynchus*, *Grampus griseus*, *Lagenodelphis hosei*, *Neophocaena asiaeorientalis*, *Stenella coeruleoalba*, and stored at −20 °C until extraction. Each 25 mg of tissues of cerebrum, cerebellum, skeletal muscle, and myocardium were homogenized after adding 180 μL saline. Twenty μL proteinase K and 10 μL of spike-in standards were added into homogenized mixtures. DNA was extracted using the automatic bead-based extraction (taco™ mini GeneReach, Lexington, MA, USA) (Figure 7). The supernatants of spiked mixtures (200 μL) were added into the first well of the extraction plate, containing lysis buffer and subjected to the extraction steps as described in the manufacturer’s user manual, resulting in 200 μL of extracted sample. All extracted samples were placed at −20 °C until used. DNA was also extracted from the spiked mixtures using the DNeasy^®^ Blood & Tissue kit (Qiagen, Valencia, CA, USA), and stored at −20 °C until used. DNA concentrations were determined using a fluorescence-based quantitation method (Qubit^®^ dsDNA BR Assay Kit on Qubit^®^ 2.0 Fluorometer (Invitrogen, Carlsbad, CA, USA). The copy number was calculated by the following formula: amount (copy/µL) = 6 × 10^23^ (copy/mol) × concentration (g/µL)/MW (g/mol).

### 2.4. Insulated Isothermal PCR

The primers and probes used in this study targeted the RE and B1 genes of *T. gondii*. The housekeeping gene of cetacean as an internal control was based on the B2M gene (GenBank accession no. DQ404542.1) [46,47] (Table 2). The fluorescence-labeled probes of RE and B1 were designed for the 520 nm (FAM), and B2M were designed for 550 nm (VIC). The insulated isothermal PCR (iiPCR) reaction conditions were tested systematically to obtain the highest sensitivity and specificity. In singleplex RE or B1 iiPCR, 45 μL of Premix Buffer B (GeneReach), 2.5 μL of 10 μM primers, 1.5 μL of 5 μM probe and 5 μL of the DNA template was added. In duplex B1/B2M iiPCR, 45 μL of Premix Buffer B, 2.5 μL of 10 μM B1 primers and B2M primers, 1.5 μL of 5 μM B1 probe, 0.375 μL of 5 μM B2M probe and 5 μL of the sample DNA template was added to the reaction. Subsequently, 50 μL of the final mixture was transferred into an R-tube™ (GeneReach), sealed with a cap, spun for 10 s in a centrifuge (cubee™, GeneReach), and placed into a dual-channel iiPCR device (POCKIT™ Micro Duo Nucleic Acid Analyzer, GeneReach) (Figure 7). Signal-to-noise (S/N) ratios, i.e., light signals collected after iiPCR/fluorescent signals collected before iiPCR, were converted automatically to “+” and “−” according to the default S/N thresholds by the built-in algorithm. 

### 2.5. Real-Time PCR 

Real-time PCR (qPCR) was performed in a volume of 10 μL with primers and probes, concentration of 0.4 μM and 0.2 μM, respectively, 5 μL of 2x QuantiNova Probe PCR Master Mix (Applied Biosystems, Foster City, CA, USA) and 2.5 μL DNA template in an Eco™ PCR system (Illumina, San Diego, CA, USA). The qPCR conditions were as follows: 2 min at 95 °C, followed by 40 cycles of 5 s at 95 °C and 5 s at 60 °C. Nuclease-free water instead of DNA extract was used as the no template control. All spiked samples, negative controls (no template control and non-spiked tissues) and positive controls were run in triplicate. Samples with cycle threshold (Ct) > 40 were considered negative.

### 2.6. Statistics

Limit of detection 95% (LoD95%) of the assay was determined by Probit analysis (a non-linear regression model) using at 95% confidence interval by using the statistical programming language R version 4.0.3 (R Core Team, 2020) package (vcd). Kappa analysis of a 2 × 2 contingency table was used to assess the agreement between two assays.

## 3. Results

### 3.1. DNA Concentration 

**Figure 8:**
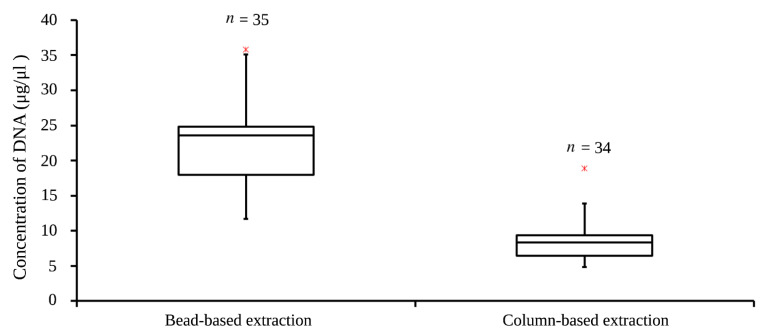
The boxplot of concentration of DNA showed the median (line), Q1 and Q3 percentiles (box), 1.5 interquartile range (whiskers) and outliers (red asterisks). The Q1 of bead-based extraction was 18.0 μg/μL and Q3 was 24.8 μg/μL. The Q1 of column-based extraction was 6.4 μg/μL and Q3 was 9.4 μg/μL.

The 25 mg of muscle tissues of cetaceans were used in column-based and bead-based extraction. The median of DNA concentration in bead-based extraction (*n* = 35, 23.6 μg/μL) was higher than that of in column-based extraction (*n* = 34, 8.3 μg/μL) (Figure 8). The average DNA concentration of bead-based extraction (21.8 μg/μL) was about 2.5 times higher than that of column-based extraction (8.7 μg/μL). The Q3-Q1 of DNA concertation in bead-based extraction (6.8 μg/μL) was more than that of in column-based (3 μg/μL), suggesting there was a wider range of DNA concentration in bead-based extraction. The DNA concentration of bead-based extraction was significantly higher than that of column-based extraction (*t*-test, *p* < 0.00001).

### 3.2. Analytical Specificity

The analytical specificity was performed using Primer-BLAST [48], with a traditional nr database. The specificity of the assay was also examined using the known DNA sequences of other closely related protozoan parasites (*Besnoitia* sp., *Cryptosporidium parvum*, *Cystoisospora*, *Echinococcus granulosus*, *Eimeria cylindrica*, *Enterocytozoon bieneusi*, *Giardia duodenalis*, *Hammondia hammondi, Neospora caninum*, *Neospora* sp., *Nephroisospora* sp., *Plasmodium falciparum*, *Sarcocystis* sp., *Trichinella spiralis*, *Trichomonas vaginalis*). The sequence of B1 primers only target *T. gondii* DNA and did not target the DNA of other sequences, ensuring high specificity for target amplification, while the RE primers had matches to *H. hammondi*.

### 3.3. Analytical Sensitivity

The analytical sensitivities of the iiPCR assays were evaluated using serial dilutions (3 × 10^7^~3 × 10^−1^ copy/μL) of spike-in standards. All extracted DNA were subsequently subjected to the iiPCR and the qPCR. Overall, 100% positive results were obtained in the iiPCR reactions containing a range from 3 × 10^7^ to 3 × 10^−1^ copy/μL of the spike-in standard (Table 3). The thirty no-template controls were all negative in both B1 and RE iiPCR. The 100% detection endpoints were 3 copy/μL in B1 and RE iiPCR, and were comparable to qPCR assays. The LOD 95% of the assay calculated by the Probit regression analysis was 2.6 copy/μL.

### 3.4. Sensitivity in Singleplex iiPCR

Three hundred copies of spike-in standard added into 25 mg of cerebrum tissue and muscle tissue, and 100% detection endpoints were confirmed (Table 4). The 100% detection endpoints of the B1 iiPCR included 300 copies in cerebrum and muscle tissues, while the 100% detection endpoints of cerebellum and myocardium tissues included 3000 copies. In comparison, the 100% detection endpoints of the B1 qPCR were for 300 copies in cerebrum, cerebellum and muscle tissues, while it was 3000 copies in myocardium tissue. The 100% detection endpoints of the RE iiPCR were for 3000 copies in four tissues. The RE qPCR was for 300 copies in cerebrum, muscle and myocardium tissues, while it was for 3000 copies in cerebellum tissue. 

### 3.5. Sensitivity in Duplex iiPCR

The clinical sensitivities of the B1/B2M iiPCR were evaluated by using different concentrations of spike-in standard and compared to that of B1 qPCR assay (Table 5). The 100% detection endpoints of the B1/B2M iiPCR were for 375 copies in 25 mg of muscle tissue, 750 copies in 25 mg of cerebrum and cerebellum tissues, while for 3000 copies in 25 mg of myocardium tissue. In contrast, the 100% detection endpoints of the B1 qPCR were for 187.5 copies for the spike-in standard in cerebrum, cerebellum and muscle tissues, while 7500 copies in myocardium tissue. Additionally, the sensitivity of qPCR was about four fold higher than B1/B2M iiPCR. The RE/B2M iiPCR was not successful in the pre-test (data not shown).

### 3.6. Performance Evaluation of the B1/B2M iiPCR

The performance of the duplex B1/B2M iiPCR was evaluated for the detection of the spike-in standard in 296 spiked samples and was compared to the B1 qPCR assay. These were comprised of 76 cerebrum tissues, 68 cerebellum tissues, 68 muscle tissues and 84 myocardium tissues from stranded cetaceans. The overall agreement between a singleplex B1 qPCR and B1/B2M iiPCR was 92% (CI 95%: 78–90%) with a kappa value of 0.84, thus confirming an almost perfect level of agreement between the duplex iiPCR and qPCR in detecting the spike-in standard in all tissues (Table 6).

#### 3.6.1. Cerebrum Tissue

Cerebrum tissues were spiked with different concentrations (3 × 10^7^, 3 × 10^6^, 3 × 10^5^, 3 × 10^4^, 3 × 10^3^, 750, 375, 187.5, 0 [mock] copies) of spike-in standard, providing a total of 76 *T. gondii*-spiked and 30 mock samples (Table 6). The B1 qPCR identified all *T. gondii*-spiked samples 3 × 10^7^ (*n* = 2), 3 × 10^6^ (*n* = 2), 3 × 10^5^ (*n* = 2), 3 × 10^4^ (*n* = 2), 3 × 10^3^ (*n* = 2), 750 (*n* = 20), 375 (*n* = 8), 187.5 (*n* = 8) copies, while none of the mock samples yielded positive results (0/30) (Table 6). The B1/B2M iiPCR detected 39 of 46 *T. gondii*-spiked samples containing 3 × 10^7^ (*n* = 2), 3 × 10^6^ (*n* = 2), 3 × 10^5^ (*n* = 2), 3 × 10^4^ (*n* = 2), 3 × 10^3^ (*n* = 2), 750 (*n* = 20), 375 (*n* = 4), 187.5 (*n* = 5) copies, while none of the mock samples yielded positive results (0/30). All *T. gondii*-spiked samples that yielded false-negative results in B1/B2M iiPCR assay (7/76) ranged from 375 (*n* = 4) copies to 187.5 (*n* = 3) copies. The B1 qPCR and the B1/B2M iiPCR assay showed an agreement of 91 % for cerebrum tissue (κ = 0.82 [CI 95%: 69–94%]).

#### 3.6.2. Cerebellum Tissue

Cerebellum tissues were spiked with different concentrations (3 × 10^7^, 3 × 10^6^, 3 × 10^5^, 3 × 10^4^, 3 × 10^3^, 750, 375, 187.5, 0 [mock] copies) of spike-in standard as previously indicated, providing a total of 68 *T. gondii*-spiked and 30 mock samples (Table 6). The B1 qPCR identified 38/68 *T. gondii*-spiked samples contained 3 × 10^7^ (*n* = 2), 3 × 10^6^ (*n* = 2), 3 × 10^5^ (*n* = 2), 3 × 10^4^ (*n* = 2), 3 × 10^3^ (*n* = 2), 750 (*n* = 20), 375 (*n* = 4), 187.5 (*n* = 4) copies, while none of the mock samples yielded positive results (0/30).The B1/B2M iiPCR detected 34/68 *T. gondii*-spiked samples contained 3 × 10^7^ (*n* = 2), 3 × 10^6^ (*n* = 2), 3 × 10^5^ (*n* = 2), 3 × 10^4^ (*n* = 2), 3 × 10^3^ (*n* = 2), 750 (*n* = 20), 375 (*n* = 2), 187.5 (*n* = 2) copies, while none of the mock samples yielded positive results (0/30). All *T. gondii*-spiked samples that yielded false negative results using the B1/B2M iiPCR assay (4/68) range from 375 (*n* = 2) to 187.5 (*n* = 2) copies. The B1 qPCR and the B1/B2M iiPCR assay showed an agreement of 94% for cerebellum tissue (κ = 0.89 [CI 95%: 77–99%]).

#### 3.6.3. Muscle Tissue 

Muscle tissues were spiked with different concentrations (3 × 10^7^, 3 × 10^6^, 3 × 10^5^, 3 × 10^4^, 3 × 10^3^, 750, 375, 187.5, 0 [mock] copies) of spike-in standard as previously indicated, providing a total of 68 *T. gondii*-spiked and 30 mock samples (Table 6). The B1 qPCR identified 38/68 *T. gondii*-spiked samples contained 3 × 10^7^ (*n* = 2), 3 × 10^6^ (*n* = 2), 3 × 10^5^ (*n* = 2), 3 × 10^4^ (*n* = 2), 3 × 10^3^ (*n* = 2), 750 (*n* = 20), 375 (*n* = 4), 187.5 (*n* = 4) copies, while none of the mock samples yielded positive results (0/30) (Table 6) The B1/B2M iiPCR detected 37/68 *T. gondii*-spiked samples contained 3 × 10^7^ (*n* = 2), 3 × 10^6^ (*n* = 2), 3 × 10^5^ (*n* = 2), 3 × 10^4^ (*n* = 2), 3 × 10^3^ (*n* = 2), 750 (*n* = 20), 375 (*n* = 4), 187.5 (*n* = 3) copies, while none of the mock samples yielded positive results (0/30). All *T. gondii*-spiked samples that yielded false-negative results using the B1/B2M iiPCR assay (1/68) contained 187.5 (*n* = 1) copies. The B1 qPCR and the B1/B2M iiPCR assays showed an agreement of 99% for muscle tissue (κ = 0.97 [CI 95%: 91–100%]).

#### 3.6.4. Myocardium Tissue

Myocardium tissues were spiked with different concentrations (3 × 10^7^, 3 × 10^6^, 3 × 10^5^, 3 × 10^4^, 3 × 10^3^, 750, 0 [mock] copies) of spike-in standard as previously indicated, leading to a total of 84 *T. gondii*-spiked and 30 mock samples (Table 6). The B1 qPCR identified 54/84 *T. gondii*-spiked samples contained 3 × 10^7^ (*n* = 2), 3 × 10^6^ (*n* = 2), 3 × 10^5^ (*n* = 2), 3 × 10^4^ (*n* = 4), 3 × 10^3^ (*n* = 20), 750 (*n* = 24) copies, while none of the mock samples yielded positive results (0/30) (Table 6). The B1/B2M iiPCR detected 42/84 *T. gondii*-spiked samples contained 3 × 10^7^ (*n* = 2), 3 × 10^6^ (*n* = 2), 3 × 10^5^ (*n* = 2), 3 × 10^4^ (*n* = 4), 3 × 10^3^ (*n* = 20), 750 (*n* = 12) copies, while none of the mock samples yielded positive results (0/30). All *T. gondii*-spiked samples that yielded false-negative results using the B1/B2M iiPCR assay (12/84) contained 750 (*n* = 12) copies of spiked DNA. The B1 qPCR and the B1/B2M iiPCR assays showed an agreement of 86% for myocardium tissue (κ = 0.71 [CI 95%: 57–86%]).

## 4. Discussion

Previously, there existed many obstacles to monitoring *T. gondii* in stranded cetaceans in some countries. One is that decomposed samples could lead to low-quality DNA, resulting in low detection rates. Another obstacle is that the long-distance transportation of samples without proper storage can also cause decomposition. The main obstacles to detecting *T. gondii* in stranded cetaceans are the lack of appropriate equipment and well-trained technicians. Therefore, many countries or regions still have less effort to detect *T. gondii* in stranded animals. The research goal of this platform was to detect *T. gondii* in stranded cetaceans. The advantages of obtaining a high DNA concentration (Figure 8) in a fraction of time on a bead-based extraction (up to 100 mg tissue per sample) can overcome the shortcomings of low-quality decomposed samples, thereby improving the sensitivity of subsequent detection. The bead-extraction machine is relatively portable (dimension: 260 (W) × 265 (D) × 300 (H) mm, 5.5 kg), which could be put in a portable pelican case. Additionally, the simple operation of beads-based extraction is easy to train the field investigators. The platform uses duplex iiPCR (520 nm for pathogen and 550 nm for internal control) designed to simultaneously detect *T. gondii* and B2M gene of cetacean on samples, which can prevent the false-negative results of pathogen detection and improve surveillance accuracy. This portable iiPCR platform combined with automatic bead-based extraction is suitable for the on-site detection of pathogens. 

In previous studies, *T. gondii* has been detected in several organs in cetaceans, including the brain, heart, skeletal muscle, mesenteric lymph nodes, liver, spleen, lung, and kidney [32,33,35,49,50]. The tissue samples from stranded cetaceans used in this study were collected from CNS, heart and skeletal muscle. The sensitivities of singleplex iiPCR using cerebrum, cerebellum, muscle and myocardium samples were around 300~3000 copies per 25-mg tissue, suggesting a sensitivity of around 8.6~86 bradyzoites per 25-mg tissue in B1 iiPCR, and around 1~10 bradyzoites per 25-mg tissue in RE iiPCR (Table 4). The sensitivities of singleplex B1 iiPCR using cerebellum and myocardium samples were lower than those of the cerebrum and muscle samples. In singleplex RE iiPCR, the muscle samples had a higher sensitivity than other organ samples (Table 4). The sensitivities of duplex B1/B2M iiPCR were around 21~86 bradyzoites per 25 mg tissue (Table 5). Both the duplex and singleplex iiPCR can detect one tissue cyst (hundreds of bradyzoites) in tissue samples, while the results indicated that four primers and two probes of B1 and B2M might interact with each other, leading to a slightly lower sensitivity in duplex detection. In a previous study of B1-LAMP assay [51], DNA from tachyzoites was maintained in Vero cells and was extracted using phenol/chloroform extraction. The sensitivity was 0.1 tachyzoites per μL, suggesting seven copies per reaction. In another study of a B1-RPA lateral flow assay [52], oocysts were repeatedly frozen and ground in liquid nitrogen and were extracted using a stool DNA kit. The sensitivity was 0.1 oocysts per reaction in water and soil samples, suggesting 28 copies per reaction. Compared to previous studies, the sensitivity of iiPCR in the present study is similar. Moreover, the overall agreement between the duplex iiPCR and qPCR was at an almost perfect level (kappa > 0.81) of agreement in detecting spike-in standard in all samples (Table 6), while the highest agreement was noticed in muscle and lowest agreement in myocardium, indicating that sample type may affect detection rate.

Primer-BLAST revealed that the B1 primers have no matches to other genes, whereas the RE primers may amplify the repetitive sequence of *H. hammondi*, which has been noticed in a previous study [53]. *H. hammondi* is an apicomplexan parasite with the cat as its definitive host. *H. hammondi* and *T. gondii* share a small group of intermediate hosts, including rodents, goats, and roe deer [54,55]. Unlike *T. gondii*, the oocysts of *H. hammondi* are infective to mice, and cats get infection only by consuming tissue cysts [54,56]. Avians are not intermediate hosts of *H. hammondi* [57]. To our knowledge, there has not been reported *H. hammondi* infection in whales and dolphins. Additionally, *H. hammondi* is less lethal than *T. gondii*, and has no disease in these hosts [53,56]. Furthermore, research on *H. hammondi* infection in cetaceans and any disease in hosts has still not been investigated. Further research on the prevalence of *H. hammondi* in cetaceans is needed.

*T. gondii*-infected felids are an important source of public health issues. The global *T. gondii* seroprevalence in domestic cats was estimated to be 35% (highest: 52% in Australia; lowest: 27% in Asia), and 59% in wild felids (highest: 67% in Europe and Asia; lowest: 45% in North America) [58]. The *T. gondii* serosurveillance conducted in China showed that the seroprevalence in cat owners (11.86%) is higher than in non-pet cat owners (7.38%), presenting a significant exposure risk to a large human population [59]. Due to the increasing prevalence of cats, this might present a significant exposure risk of humans and other animals. On a global scale, the high frequency of marine-mammal infection by land-based protozoa provides a great illustration of the connection between land and sea, and between humans and other organisms [27]. Because humans consume similar prey, the risk of human exposure and the adverse health effects of the protozoal parasite loading of marine ecosystems are likely to increase. Coastal urban development, agricultural intensification, and the increasing manipulation of river flow into oceans can facilitate environmental contamination and parasite spread into marine ecosystems. It is speculated that many *T. gondii* oocysts enter the marine system and infect the marine mammals in Asia. Still, more evidence of toxoplasmosis in cetaceans in Asia is needed. 

Several studies reported correlations between protozoal disease in marine mammals and immunosuppressive factors, including morbilliviruses, anthropogenic pollutants, and bacterial sepsis [60,61,62]. On the basis of this evidence, pollution and other stress factors around the nearby ocean may occur. The monitoring of *T. gondii* is important to the epidemiological patterns associated with stress and immunosuppression and an indicator of ocean health. Additionally, climate and habitat change can impact protozoan pathogen exposure, infection, and disease outcome [63,64]. These findings further emphasize the importance of pathogen spread between definitive terrestrial hosts and sympatric marine mammals. The *T. gondii*-iiPCR platform developed in this study could be used to better understand land-to-sea coastal oocyst pollution threatening both marine ecosystem and human beings. This platform provides quick results (~1.5 h) with a field-deployable system and is suitable for on-site diagnosis to monitor the quality of the aquatic ecosystem’s health. It may help to develop new strategies to improve the environment via the warning of sentinel animals. This platform could also be applied to other important pathogens for which information is lacking, in cetaceans, such as *Brucella ceti* and morbillivirus in stranded cetaceans, providing an approach to evaluating aquatic ecosystem health and gaining early warnings about negative impacts on humans and animals.

## Figures and Tables

**Table 1:** *T. gondii* or *T. gondii*-like protozoan parasite exposure in cetaceans [27,31,32,33,34].

Family	Genus	Species
Balaenopteridae	*Balaenoptera*	Blue whale (*Balaenoptera musculus*)
Balaenopteridae	*Balaenoptera*	Bryde’s whale *(Balaenoptera edeni)*
Balaenopteridae	*Balaenoptera*	Fin whale (*Balaenoptera physalus*)
Balaenopteridae	*Balaenoptera*	Minke whale (*Balaeneoptera acutorostrata*)
Balaenopteridae	*Balaenoptera*	Sei whale (*Balaenoptera borealis*)
Balaenopteridae	*Megaptera*	Humpback whale (*Megaptera novaeangliae*)
Delphinidae	*Cephalorhynchus*	Hector’s dolphin (*Cephalorhynchus hectori*)
Delphinidae	*Delphinus*	Common dolphin (*Delphinus delphis*)
Delphinidae	*Feresa*	Pygmy killer whale *(Feresa attenuata)*
Delphinidae	*Globicephala*	Pilot whale *(Globicephala macrorhynchus)*
Delphinidae	*Globicephala*	Pilot whale (*Globicephala melaena*)
Delphinidae	*Grampus*	Risso’s dolphin (*Grampus griseus*)
Delphinidae	*Lagenodelphis*	Fraser’s dolphin *(Lagenodelphis hosei)*
Delphinidae	*Lagenorhynchus*	White-sided dolphin (*Lagenorynchus obliquidens*)
Delphinidae	*Orcinus*	Killer whale (*Orcinus orca*)
Delphinidae	*Peponocephala*	Melon-headed whale *(Peponocephala electra)*
Delphinidae	*Pseudorca*	False killer whale (*Pseudorca crassidens*)
Delphinidae	*Sotalia*	Guiana dolphin (*Sotalia guianensis*)
Delphinidae	*Sousa*	Humpbacked dolphin (*Sousa chinensis*)
Delphinidae	*Stenella*	Pantropical spotted dolphin *(Stenella attenuata)*
Delphinidae	*Stenella*	Spinner dolphin (*Stenella longirostris*)
Delphinidae	*Stenella*	Spotted dolphin (*Stenella frontalis*)
Delphinidae	*Stenella*	Striped dolphin (*Stenella coeruleoalba*)
Delphinidae	*Steno*	Rough-toothed dolphin *(Steno bredanensis)*
Delphinidae	*Tursiops*	Bottlenose dolphin (*Tursiops aduncus*)
Delphinidae	*Tursiops*	Bottlenose dolphin (*Tursiops truncatus*)
Iniidae	*Inia*	Amazon River dolphin (*Inia geoffrensis*)
Kogiidae	*Kogia*	Pygmy sperm whale (*Kogia breviceps*)
Monodontidae	*Delphinapterus*	Beluga (*Delphinapterus leucas*)
Monodontidae	*Monodon*	Narwhal (*Monodon nonoceros*)
Phocoenidae	*Neophocaena*	Finless porpoise (*Neophocaena phocaenoides*)
Phocoenidae	*Phocoena*	Harbor porpoise (*Phocoena phocoena*)
Physeteridae	*Physeter*	Sperm whale (*Physeter macrocephalus*)
Ziphiidae	*Mesoplodon*	Beaked whale (*Mesoplodon* sp.)

**Table 2:** The sequence of primers and probes.

Primers and Probes	Nucleotide Sequences (5′–3′)	Target Genes	Function	Amplicons	References
RE-R	5′-TCCTCACCCTCGCCTTCAT-3′	529-bp repeat element	Unknown function	60 bp	[45]
RE-F	5′-GCTCCTCCAGCCGTCTTG-3′				
RE-P	5′-6-FAM-AGGAGAGATATCAGGACTGTA -MGB-NFQ -3				
B1-R	5′-AGACTGTACGGAATGGAGACGAA-3′	B1 gene	cGMP dependent protein kinase activity	61 bp	[44]
B1-F	5′-GCATTGCCCGTCCAAACT-3′				
B1-P	5′-6-FAM–CAACAACTGCTCTAGCG–MGB-NFQ-3′				
B2M-R	5′- GCGTTGGGAGTGAACTCAG-3′	B2M gene	β2-microglobulin	78 bp	[46,47]
B2M-F	5′-GGTGGAGCAATCAGACCTGT-3′				
B2M-P	5′-VIC-TCAGCAAGGACTGGTCTT-MGB-NFQ-3′				

**Table 3:** Analytical sensitivity of singleplex iiPCR.

Standard (Copy/μL)	RE qPCR No. Positive/ No. Tested	Rate (%)	RE iiPCR No. Positive/ No. Tested	Rate (%)	B1 qPCR No. Positive/ No. Tested	Rate (%)	B1 iiPCR No. Positive/ No. Tested	Rate (%)
3 × 10^7^	2/2	100	2/2	100	2/2	100	2/2	100
3 × 10^6^	2/2	100	2/2	100	2/2	100	2/2	100
3 × 10^5^	2/2	100	2/2	100	2/2	100	2/2	100
3 × 10^4^	4/4	100	4/4	100	4/4	100	4/4	100
3 × 10^3^	4/4	100	4/4	100	4/4	100	4/4	100
3 × 10^2^	4/4	100	4/4	100	4/4	100	4/4	100
3 × 10^1^	4/4	100	4/4	100	4/4	100	4/4	100
3 × 10^0^	4/4	100	4/4	100	4/4	100	4/4	100
3 × 10^−1^	2/4	50	2/4	50	2/4	50	2/4	50

**Table 4:** Sensitivity in singleplex iiPCR.

Samples from Different Organs	Standard in 25 mg of Tissues (Copy)	B1 iiPCR No. Positive/ No. Tested	B1 qPCR No. Positive/ No. Tested	RE iiPCR No. Positive/ No. Tested	RE qPCR No. Positive/ No. Tested
Cerebrum	3 × 10^3^	4/4	4/4	4/4	4/4
	3 × 10^2^	4/4	4/4	1/4	4/4
	3 × 10^1^	0/4	1/4	0/4	1/4
	3 × 10^0^	0/4	1/4	0/4	0/4
Cerebellum	3 × 10^3^	4/4	4/4	4/4	4/4
	3 × 10^2^	2/4	4/4	2/4	3/4
	3 × 10^1^	2/4	3/4	0/4	0/4
	3 × 10^0^	0/4	0/4	0/4	0/4
Muscle	3 × 10^3^	4/4	4/4	4/4	4/4
	3 × 10^2^	4/4	4/4	4/4	4/4
	3 × 10^1^	0/4	3/4	0/4	3/4
	3 × 10^0^	0/4	0/4	0/4	0/4
Myocardium	3 × 10^3^	4/4	4/4	4/4	4/4
	3 × 10^2^	2/4	3/4	2/4	4/4
	3 × 10^1^	0/4	0/4	1/4	3/4
	3 × 10^0^	0/4	0/4	0/4	1/4

**Table 5:** Sensitivity in duplex iiPCR.

Samples from Different Organs	Standard in 25 mg of Tissues (Copy)	B1/B2M iiPCR No. Positive/ No. Tested	Rate (%)	B1 qPCR No. Positive/ No. Tested	Rate (%)
Cerebrum	3 × 10^7^~3 × 10^3^	10/10	100	10/10	100
	750	20/20	100	20/20	100
	375	4/8	50	8/8	100
	187.5	5/8	62.5	8/8	100
Cerebellum	3 × 10^7^~3 × 10^3^	10/10	100	10/10	100
	750	20/20	100	20/20	100
	375	2/4	50	4/4	100
	187.5	2/4	50	4/4	100
Muscle	3 × 10^7^~3 × 10^3^	10/10	100	10/10	100
	750	20/20	100	20/20	100
	375	4/4	100	4/4	100
	187.5	3/4	75	4/4	100
Myocardium	3 × 10^7^~3 × 10^3^	30/30	100	10/10	100
	750	12/24	50	24/24	100

**Table 6:** Performance evaluation of the B1/B2M iiPCR using *T. gondii*-spiked cetacean samples.

Samples from Different Organs	B1/B2M iiPCR	B1 qPCR ^a^			Agreement (κ, CI95%) ^b^
		Positive	Negative	Total	
Overall performance	Positive	152	0	152	92% (0.84[0.78–0.90])
	Negative	24	120	144	
	Total	176	120	296	
Cerebrum	Positive	39	0	39	91% (0.82[0.69–0.94])
	Negative	7	30	37	
	Total	46	30	76	
Cerebellum	Positive	34	0	34	94% (0.89[0.77–0.99])
	Negative	4	30	34	
	Total	38	30	68	
Muscle	Positive	37	0	37	99% (0.97[0.91–1.00])
	Negative	1	30	31	
	Total	38	30	68	
Myocardium	Positive	42	0	42	86% (0.71[0.57–0.86])
	Negative	12	30	42	
	Total	54	30	84	

^a^ Samples that yielded inconclusive results (Ct > 40) were not included in the analysis. ^b^ The Kappa statistic and 95% confidence interval is shown within brackets.

## Data Availability

Not applicable.
